# Associations Between Perceived Stress and Cortisol Biomarkers in Predominantly Latino Adolescents

**DOI:** 10.21203/rs.3.rs-5227487/v1

**Published:** 2024-11-15

**Authors:** Fatimata Sanogo, Avaion Ruth, Victoria K. Cortessis, Li Ding, Richard M. Watanabe, Marc J. Weigensberg

**Affiliations:** Keck School of Medicine of USC; Keck School of Medicine of USC; Keck School of Medicine of USC; Keck School of Medicine of USC; Keck School of Medicine of USC; USC Diabetes and Obesity Research Institute

**Keywords:** Perceived stress, cortisol, Latino Adolescents

## Abstract

**Objective:**

The relationship between cortisol and perceived stress is poorly understood. We sought to determine whether perceived stress is associated with cortisol biomarkers in adolescents.

**Methods:**

We examined 229 adolescents (mean age = 15.8 years). We measured perceived stress with the 14-item Perceived Stress Scale (PSS) questionnaire, serum cortisol (sCOR), salivary Cortisol Awakening Response (CAR: 30-minute post-awakening - awakening), salivary Diurnal Cortisol Slope (DCS: evening-awakening), and total daily salivary cortisol (TDC). We used multivariable linear regression to estimate baseline associations between PSS, TDC, sCOR, and FBG. We used mixed effects linear regression to estimate baseline associations between PSS and CAR and DCS. We tested twelve-week longitudinal associations between PSS and cortisol biomarkers using random effects regression. Analyses were adjusted for age, gender, and BMI.

**Results:**

There were statistically significant associations between PSS and TDC (beta= −104.36 ± 34.3; p = 0.002) at baseline and between PSS and DCS at 12 weeks (beta= −0.058 ± 0.02; p = 0.01), but no association between PSS and sCOR or CAR (p > 0.26 for all) at baseline or 12-weeks. There were no associations between change in PSS and change in cortisol biomarkers longitudinally.

**Conclusion:**

In adolescents, perceived stress measured by the PSS was inconsistently associated with TDC and DCS, and consistently unassociated with CAR and sCOR. Studies reporting on PSS outcomes should exercise caution when making conclusions about cortisol biomarkers. There’s a need for an instrument that captures a global measure of perceived stress and is sensitive to HPA functioning.

## Introduction

The human stress response is regulated by two major physiological systems: the autonomic nervous system and the hypothalamic-pituitary-adrenal (HPA) axis^1^. The steroid hormone cortisol is involved in physiological processes including metabolism and HPA-related stress response^2,3^. Daily HPA axis activity can be represented by diurnal cortisol patterns that are typically assessed as the cortisol awakening response (CAR), diurnal cortisol slope (DCS)^4,5^, or total daily salivary cortisol (TDC). CAR is the increase in salivary cortisol levels within 30–60 min after awakening in the morning, DCS is the decrease in salivary cortisol from morning to evening over the waking day^5^, and TDC provides an integrated measure of cortisol secretion and is commonly quantified by calculating the area under the time-cortisol curve. Measures of cortisol also include serum cortisol (sCOR), typically measured first thing in the morning^6^.

Individual self-rating of the stressfulness of events is generally a better predictor of health outcomes than objective measures of stressful life events^7,8^. The Perceived Stress Scale (PSS) quantifies subjective stress in a general sense; i.e., the degree to which one’s life is perceived to be unpredictable, uncontrollable, and/or overloading^9^ over the preceding month. Important gaps remain in understanding the relationship between subjective stress, particularly as measured by PSS, and cortisol biomarkers. Studies examining the association between chronic subjective stress and cortisol biomarkers have yielded inconsistent results, whether they assessed PSS ^10–13^ or used other instruments ^14–18^ to measure subjective stress.

Further, studies investigating the association between perceived stress and cortisol biomarkers in Latino adolescents are nearly non-existent despite this understudied population being at particular risk for stress-related health outcomes^19^ including obesity^20^, obesity-related comorbidities^20,21^, type 2 diabetes^22^, and mental health issues^23,24^. Our primary objective was to determine whether in this population subjective stress, as measured by the PSS, is associated with cortisol biomarkers as measured by sCOR, CAR, DCS, and TDC.

## Methods

### Study Design and Participants

We used original data on PSS and levels of cortisol assessed among participants in the Imagine HEALTH (Healthy Eating Active Living Total Health) study ^25^, a randomized controlled trial that tested a 12-week lifestyle education program combined with the mind-body modality of guided imagery to address obesity-related lifestyle behaviors and stress biomarkers in adolescents. Specific details of the intervention protocol, guided imagery delivery and content, and primary outcomes have been previously described^26,27^. The nature of the trial and intervention have no direct relevance to the analyses presented here, but we briefly provide study details here for clarity. The study included 229 predominantly Latino male and female high school students who attended up to 3 after-school classes per week for the 12-week intervention. Those with chronic illness (including diabetes), cognitive behavioral disability, or prior diagnosis of clinical eating disorder or psychiatric disorder and those taking medications known to affect the HPA axis (*e.g.*, glucocorticoids) were excluded. Participants were cluster-randomized on school level into four intervention arms: non-intervention control, lifestyle education alone, lifestyle education plus stress reduction guided imagery, and lifestyle education plus lifestyle behavior guided imagery. The study was approved by the Institutional Review Board of the University of Southern California. Informed consent was obtained from parents and youth assent from participants. All methods were performed in accordance with the relevant guidelines and regulations.

### Measurement Visits

Measurement visits were conducted on weekend mornings at baseline and after the 12-week intervention. Participants arrived following an overnight fast and fasting blood was drawn between 7:30 and 9:30 AM for assessment of sCOR. Perceived stress was measured using the 14-item PSS questionnaire^9^ administered by trained research staff, which assesses the perception of stress in the preceding month. Age, gender, ethnicity, and race were self-reported. Height and weight were measured by a stadiometer and clinical scale and used to calculate BMI.

### Self-Collected Samples and Biospecimen Processing

In the week following measurement visits, saliva samples were collected by participants in their home environments using salivettes by Salimetrics on three consecutive weekdays at each of three specific times: upon awakening, 30 minutes after awakening, and just before bedtime as previously described^27^. The timing of sample collection was assessed using the timestamp of the uploaded pictures by participants using the Zemi application^19^. Samples were kept in participants’ freezers until retrieved by study personnel.

Salivette samples were centrifuged, and saliva supernatant was aliquoted and stored in cryovials at −80°C until assayed for cortisol. A total of 993 salivary cortisol samples were available for the calculation of CAR and 972 for the calculation of DCS and TDC. No individual samples were excluded for being outliers, defined as over 3 SD from the mean. The timing of salivary sampling was 32 ± 12.4 min (n = 700) for CAR and 14.7 ± 2.8 hours (n = 596) for DCS. Twenty-five samples were excluded due to the time of the awakening sample being documented as being before 4 AM or after noon. Fasting blood samples were processed using a commercially available ELISA (Alpco; inter-assay CV = 3.8% [high], 8.1% [low]) to measure sCOR, and salivary cortisol was assessed using the Salimetrics, Inc. ELISA (inter-assay CV = 3.75% [high], 6.41% [low]). Assays were performed in the USC Diabetes and Obesity Research Institute Metabolic Lab.

### Statistical Analyses

Data from all participants were pooled for analysis because individual interventions are not pertinent to the hypotheses examined in our present study. CAR was estimated using the mean level increase method^5,25^ as the difference between 30 minutes post-awakening and awakening of salivary cortisol, and the DCS as the difference between evening and awakening salivary cortisol. TDC was computed as the total area under the curve (AUC) for the 3 daily samples using the trapezoid rule^28^. CAR, DCS, and TDC at baseline and 12 weeks were estimated by averaging the values measured at each time point.

Characteristics of study participants were summarized as means and standard deviations of continuous variables and as counts and proportions of categorical variables. We used multivariable linear regression to estimate baseline associations for single measure variables, mixed effect linear models to estimate baseline associations for repeated measure variables, and generalized least square for linear random effects regression to estimate 12-week longitudinal associations (i.e., change in PSS compared to change in cortisol measures over time). Baseline, 12-week, and longitudinal associations were evaluated for PSS and each of sCOR, CAR, DCS, and TDC. All analyses were adjusted for age, gender, and BMI. We repeated the baseline and 12-week analyses to account for potential biases due to the loss to follow-up of participants who did not complete both the baseline and 12-week assessments. The intervention in the original Imagine Health Study is not relevant to our present study, nonetheless, we conducted a mediation analysis using the classical approach^29^ in which we examined any potential influence of the intervention on the longitudinal associations between perceived stress and cortisol biomarkers. All analyses were conducted using Stata 16 (College Station, TX).

## Results

Table 1 shows the characteristics of the study participants in all intervention groups (n = 229). Mean age was 15.8 ± 0.7 yrs., 67% were female, and 95% self-identified as Hispanic or Latino. Mean BMI was 23.6 ± 7.4 kg/m^2^ with 40% categorized as either overweight (85th −94th percentile) or obese (> 95th percentile). Group mean values for PSS and cortisol biomarkers were not different at baseline and 12 weeks. Supplemental Table 1 provides the participant characteristics stratified by intervention arm.

Table 2 provides estimates of cross-sectional associations between PSS and cortisol measures at baseline and 12 weeks, as well as longitudinal change over the 12 weeks. There was a statistically significant inverse association between PSS and TDC at baseline (beta= −104.36 ± 34.3; p = 0.002), but no significant association at 12-weeks or between change in PSS and change in TDC (all p-values > 0.12) over the subsequent 12-weeks. There was a significant association between PSS and DCS at 12 weeks (beta= −0.058 ± 0.02; p = 0.01), but no significant association at baseline or between change in PSS and DCS (all p-values > 0.12) over 12 weeks. There were no significant associations between PSS and CAR, or sCOR at baseline 12 weeks, or longitudinally (p > 0.26 for all).

Supplemental Table 1 shows the results from the mediation analyses we conducted examined any potential influence of the intervention on the longitudinal associations between perceived stress and cortisol biomarkers. Our findings indicate that the intervention did not have a mediating effect.

## Discussion

The PSS is one of the most widely used tools to assess psychological stress in people over 12 years of age^30^ across different gender, racial, ethnic, and linguistic groups^31^. Therefore, characterizing the relationship of PSS levels to contemporary biological measures of stress could provide needed insight into physiologic components of the stress response that may correspond to the psychological dimension of stress captured by PSS. It is critical to have an instrument that captures a psychological dimension of subjective stress and reflects the activity of the HPA-axis for advancing integrative medicine and whole-person health research.

In cross-sectional data assessed at baseline, we found PSS to be associated with TDC, an integrated measure of physiological stress and HPA activity. The negative direction of the association indicates that, in predominantly Latino adolescents, higher perceived stress scores are related to lower TDC, as previously observed in adults^32^. This finding could suggest that PSS, as a proxy measure, could reflect the activity of the HPA-axis. However, the absence of this association at the 12-week follow-up or in longitudinal analyses suggests that the PSS may not consistently predict TDC in adolescents over time. We observed a similar pattern in the association between PSS and DCS, with a significant relationship at the 12-week follow-up that was not present at other time points in the study. Further, there was no significant association between PSS and CAR, or sCOR. This further highlights the inconsistency of the PSS in predicting cortisol biomarkers, as well as the complexity of HPA axis dynamics. The lack of association between PSS and DCS at enrollment accords with a prior study of predominantly White children and adolescents in early and mid-stage adrenarche^13^, while our findings that PSS is not associated with CAR and DCS accords with results of a cross-sectional study of female adults with fibromyalgia^10^. However, the findings of the small number of additional studies that evaluated the relationship between PSS and CAR, DCS, or sCOR are inconsistent. For example, in a study of female undergraduate students, individuals with higher mean PSS scores exhibited significantly steeper DCS compared to those with lower mean PSS scores, but CAR was similar between the two groups^11^. In another study, PSS and CAR were both higher in male students two days before an exam compared to those not taking an exam^12^, though the association between PSS and CAR was not formally evaluated. Similarly, a cross-sectional study reported significantly higher PSS and sCOR in police officers -- an occupation susceptible to high stress -- compared to their counterparts in the general population^33^, although the relationship between PSS and sCOR levels was not assessed.

In our longitudinal data comparing values of each participant’s PSS and cortisol measures assessed at enrollment to values measured 12 weeks later, change in PSS was not associated with the change in any measure of cortisol. However, the values of the individual measures changed little over this interval. The longitudinal findings therefore provide little information regarding whether change in PSS would capture changes in one or more of the cortisol measures over time if change in stress were greater. In light of the present findings, investigators who report on PSS outcomes should exercise caution when making conclusions about possible underlying cortisol biomarkers. Our findings highlight the need for an instrument that captures subjective psychological stress and reflects HPA-axis activity.

These findings contribute to accruing information regarding the complexity of the stress response and the role of cortisol. The American Psychological Association defines stress as the physiological and psychological response to a condition that threatens or challenges a person and requires some form of adaptation or adjustment^34^. Three broad classifications of stress are addressed in clinical research: environmental (objective stressors or life events), psychological (subjective appraisal and affective reactions), and biological^35^. Some previous studies assessed the relationship between subjective and objective stress in adults using either a stress biomarker other than cortisol or an instrument other than PSS to measure psychological stress. Föhr and colleagues found a positive correlation between the PSS and heart rate variability^36^. Investigating stress acutely induced by exposure to socially evaluated cold pressor test, a combination of somatic and psychosocial stressors, Buzgoova *et al*. reported higher stress perception to be correlated with lower values of a single measure of salivary cortisol^32^. Most studies that investigated the association between CAR and psychological stress used instruments that measure specific components of stress rather than the integrated measures of stress queried by the PSS. Such studies have shown associations between CAR and psychological factors including fatigue, burnout, exhaustion^37^, lack of social recognition^38^, overload^39^, financial strain^40^, employment-related stress^41^, and early life adversity^42^. These findings suggest that specialized instruments for measuring psychological stress may capture the physiological changes in CAR more effectively than the PSS. Similar to studies in adults, studies of the association between perceived stress and cortisol in adolescents and children have used instruments other than PSS and have produced mixed results. A study of adolescents identified a positive correlation between acute stress reactivity, assessed by the Groningen Social Stress Test, and mean salivary cortisol^18^, suggesting adolescents may be more reactive to acute social stress. However, a study by Maldonado et. al in children 6–12 years of age found that participants with high daily perceived stress had lower morning serum cortisol at awakening but displayed higher cognitive performance assessment for speed of memory^15^.

Cortisol plays a crucial role in the body’s stress response and is associated with both positive and negative physiological responses^43^. While prevailing perspectives often portray stress as fundamentally detrimental, contemporary psychological frameworks propose that stress is not inherently maladaptive; a growing body of literature suggests it can have positive and desirable outcomes^44^. Positive stress, often referred to as “eustress,” is a psychological concept that describes such stress as beneficial, in contrast to distress, which is perceived as negative and detrimental to health and wellness^45^. In adolescents, total daily cortisol was previously shown to be associated with higher levels of pubertal development; 23% higher for every point increase on the five-point pubertal development scale, consistent with the notion that positive stress is associated with growth and development^17^. Additionally, a study among undergraduate students showed measures of eustress to be associated with life satisfaction, hope, and self-efficacy^46^. Our study was not designed to disentangle the two aspects of stress. The PSS does not deliberately differentiate between distress and eustress, which may explain the mixed and inconsistent results. In light of the modest existence of the literature, reviewed in full here, future studies should aim to distinctly capture both eustress and distress, evaluate their correlation with the PSS, and subsequently analyze their relationship with cortisol biomarkers

This research had several strengths. The Imagine HEALTH Study provided an opportunity to explore associations between PSS and cortisol biomarkers in adolescents. There is a paucity of research on PSS and cortisol in this age group, and Latino adolescents are particularly underrepresented in research despite being at increased risk for adverse health outcomes that may be related to stress and obesity-related disease^20–22^. To our knowledge, this is the first study to report both cross-sectional and longitudinal findings on the relationship between PSS and cortisol biomarkers in predominantly Latino adolescents. The longitudinal structure of the data provided opportunities to explore a dynamic relationship between PSS and cortisol biomarkers over time, allowing a more nuanced examination of stress relationships. Moreover, mediation analyses show that the findings reported are not explained by the intervention group in which study participants were randomized in the original Imagine HEALTH Study.

Limitations of this study include that TDC was computed using three measures of cortisol per day on each of three consecutive days, rather than by the usual standard of five daily measures^4^. Nonetheless, Hartville and colleagues showed that the TDC derived from 15 samples over three days correlates with R of approximately 0.69 with that derived from only three samples assessed for a single day^47^, suggesting that while not optimal, a three-sample protocol still provides a meaningful estimate of daily cortisol exposure.

In conclusion, our study revealed inconsistent associations between perceived stress and both total daily salivary cortisol and diurnal cortisol slope at different times. We found no evidence of an association between PSS with either sCOR or CAR, and no significant longitudinal relationships between changes in PSS and changes in cortisol biomarkers. There is a clear need for an instrument that captures a global measure of perceived stress and reflects HPA-axis activity to advance integrative health and whole-person research.

## Supplementary Material

Supplement 1

## Figures and Tables

**Table 1:** Demographic and Clinical Characteristics of Participants Continuous variables are reported as mean SD and categorical variables as count (%)

		N = 229
Grade	*10th*	84 (36.7%)
	*11th*	145 (63.3%)
Gender	*Female*	153 (66.8%)
	*Male*	76 (33.2%)
Ethnicity	*Hispanic or Latino*	217 (94.7%)
	*Not Hispanic or Latino*	12 (5.3%)
Race	*White*	205 (89.9%)
	*Other*	24 (10.1%)
Body Mass Index Category	Underweight (< 5th percentile)	6 (3%)
	Normal (5th −85th percentile)	115 (50%)
	Overweight (85th −95th percentile)	41 (18%)
	Obese (> 95th percentile)	50 (22%)
		Mean ± SD
Age (years)		15.75 ± 0.66
Body Mass Index (Kg/m^2^)		23.64 ± 7.42
Perceived Stress Score	*Baseline*	24.5 ± 1.5
	*3 months*	24.5 ± 1.4
Salivary Cortisol (nmol/L)		
Baseline	*Awakening*	9.2 ± 0.46
	*+ 30 minutes*	14.9 ± 1.2
	*Evening*	2.4 ± 0.46
3 Month	*Awakening*	9.2 ± 1.0
	*+ 30 minutes*	14.4 ± 1.2
	*Evening*	2.2 ± 0.91
Cortisol Awakening Response (CAR) (nmol/L)	*Baseline*	5.2 ± 6.0
	*3 months*	4.9 ± 5.8
Diurnal Cortisol Slope (DCS) (nmol/L)	*Baseline*	−6.7 ± 5.1
	*3 months*	−7.02 ± 4.2
Total Daily Cortisol (TDC) (nmol/l)^2^	*Baseline*	7399.7 ± 3385.5
	*3 months*	7119.4 ± 3171.9
Serum Cortisol (nmol/L)	*Baseline*	409.69 ± 158.08
	*3 months*	397.12 ± 148.89

**Table 2:** Associations between PSS and Cortisol Measures Assessed as Biomarkers of Stress

	Cross-Sectional Associations at Baseline				Cross-sectional Associations at 12 Weeks				Longitudinal Associations of Changes from Baseline to 12-Weeks (N = 136)	
	Participants with Baseline Data (N_0_ = 229)		Participants with Baseline and 12 Week Data (N = 136)		Participants with 12 Week Data (N = 143)		Participants with Baseline and 12 Week Data (N = 136)			
	Beta ± SE	p-value	Beta ± SE	p-value	Beta ± SE	p-value	Beta ± SE	p-value	Beta ± SE	p-value
TDC	−105.5 ± 34.3	**0.002**	−148.5 ± 44.5	**0.001**	15.68 ± 34.13	0.65	16.33 ± 35.0	0.64	−39.44 ± 25.0	0.12
CAR	0.07 ± 0.06	0.26	−0.07 ± 0.03	0.05	0.003 ± 0.05	0.93	0.01 ± 0.03	0.79	−0.02 ± 0.05	0.60
DCS	−0.04 ± 0.05	0.38	−0.01 ± 0.03	0.71	−0.058 ± 0.02	**0.01**	−0.06 ± 0.02	**0.004**	−0.06 ± 0.04	0.12
sCOR	−1.42 ± 1.68	0.40	−1.49 ± 2.05	0.47	−1.12 ± 1.80	0.53	−1.29 ± 1.90	0.50	−1.24 ± 1.30	0.34

Data indicate mean ± SEM for each variable.

Adjusted for age, sex and body mass index.

## Data Availability

The datasets used and/or analyzed during the current study are available from the corresponding author upon reasonable request.
