# Supplementary material for: Associations Between Perceived Stress and Cortisol Biomarkers in Predominantly Latino Adolescents
Source: Res Sq. 2024 Nov 15:rs.3.rs-5227487. Preprint. [Version 1] doi: 10.21203/rs.3.rs-5227487/v1 (PMC11601828; doi:10.21203/rs.3.rs-5227487/v1)
Supplement: Supplement 1 [file NIHPPRS5227487V1-supplement-1.pdf]

Supplementary Files

This is a list of supplementary files associated with this preprint. Click to download.

- supptabl2.docx
- supptable.docx
